# Associations Between Oxidative Stress, Inflammation, Muscle Mass, and Sarcopenia Phenotypes in Older Adults with End-Stage Knee and Hip Osteoarthritis: A Cross-Sectional Study

**DOI:** 10.3390/geriatrics11050142

**Published:** 2026-10-07

**Authors:** Maria Spanoudaki, Constantinos Giaginis, Antonios Cheimaras, Stavros Pellios, Dafni Sela, Agathi Pritsa, Ioannis Moschos, Eirini Koidou, Sousana K. Papadopoulou

**Affiliations:** 1 Department of Nutritional Sciences and Dietetics, School of Health Sciences, International Hellenic University, 57001 Thessaloniki, Greece; 2 Department of Food Science and Nutrition, School of Environment, University of Aegean, Myrina, 81400 Lemnos, Greece; 3 First Orthopedic Clinic, 424 Military Hospital, 56429 Thessaloniki, Greece; 4 Department of Nutritional Sciences and Dietetics, School of Health Sciences, Hellenic Mediterranean University, 72300 Crete, Greece; 5 Department of Nursing, School of Health Sciences, International Hellenic University, 57001 Thessaloniki, Greece; 6 School of Physical Education and Sports Science, Faculty of Physical Education and Sport Sciences, Aristotle University of Thessaloniki, 62122 Serres, Greece

**Keywords:** knee osteoarthritis, hip osteoarthritis, sarcopenia, EWGSOP2, oxidative stress, inflammation, muscle mass, muscle strength, physical performance

## Abstract

**Background**: Skeletal muscle impairment is common in older adults with end-stage knee and hip osteoarthritis (OA) and contributes to disability and functional decline. Τhe relative contributions of muscle mass, muscle strength, systemic inflammation and oxidative stress to sarcopenia phenotypes in this population remain incompletely understood. This study investigated the associations between muscle-related parameters, inflammatory and oxidative stress biomarkers, and sarcopenia phenotypes in older adults with end-stage OA. **Methods**: This cross-sectional study included 88 older adults scheduled for primary total knee (*n* = 56) or total hip arthroplasty (*n* = 32). Body composition, muscle strength, physical performance, and biochemical markers were assessed using standardized procedures. Systemic inflammation was evaluated using C-reactive protein (CRP) and the neutrophil-to-lymphocyte ratio (NLR), whereas oxidative stress was assessed by urinary F_2_-isoprostanes. Sarcopenia status was categorized according to the European Working Group on Sarcopenia in Older People 2 (EWGSOP2) criteria. **Results**: Overall, 29.5% of participants were classified as having probable/confirmed sarcopenia phenotypes, with similar prevalence in knee and hip OA. Sarcopenic participants exhibited significantly lower appendicular skeletal muscle mass (ASMM), reduced handgrip strength, slower gait speed, poorer physical performance and higher body fat percentage than those without sarcopenia. Muscle strength demonstrated stronger associations with physical performance than muscle mass and remained an independent predictor of Short Physical Performance Battery (SPPB) score after adjustment for age, gender and muscle mass. Higher urinary F_2_-isoprostane concentrations were independently associated with lower ASMM after adjustment for demographic characteristics and inflammatory biomarkers, whereas CRP and NLR were not independently associated with muscle-related outcomes in the fully adjusted models. **Conclusions**: Nearly one-third (29.5%) of older adults with end-stage knee and hip OA were classified as having probable or confirmed sarcopenia according to the EWGSOP2 criteria. Oxidative stress was independently associated with skeletal muscle mass, whereas CRP and NLR were not independently associated with muscle-related outcomes. These findings highlight the relevance of muscle strength and oxidative stress in older adults with end-stage OA and warrant further longitudinal investigation.

## 1. Introduction

Osteoarthritis (OA) is the most prevalent chronic joint disorder and one of the leading causes of pain, disability, and loss of independence among older adults worldwide. Its global burden continues to increase because of population ageing and the rising prevalence of obesity, placing considerable pressure on healthcare systems [1,2]. Although OA has traditionally been regarded as a disease primarily affecting articular cartilage, it is now recognized as a whole-joint disorder involving the synovium, subchondral bone, ligaments, periarticular tissues, and skeletal muscle [1,2]. Consequently, the clinical manifestations of OA extend beyond joint degeneration to include progressive impairments in skeletal muscle function, mobility, and overall physical performance.

The relationship between OA and skeletal muscle impairment is complex and bidirectional. Persistent pain, reduced physical activity, altered biomechanics, and movement avoidance contribute to muscle atrophy and neuromuscular dysfunction, thereby compromising joint stability and functional capacity. Conversely, muscle weakness may contribute to abnormal joint loading and disease progression. Individuals with knee OA consistently exhibit marked quadriceps weakness associated with reduced mobility and poorer functional outcomes [3], whereas patients with advanced hip OA demonstrate substantial reductions in muscle strength and endurance compared with age-matched healthy individuals [4]. Importantly, declines in muscle strength do not necessarily parallel reductions in muscle mass. During ageing, muscle strength declines more rapidly than muscle quantity, a phenomenon known as dynapenia, which has emerged as a stronger predictor of disability, falls, frailty, and loss of independence than muscle mass alone [5]. Furthermore, the combined deterioration of muscle strength, muscle quality, and physical performance is associated with adverse clinical outcomes, including functional decline, hospitalization, and increased mortality [6,7].

The revised European Working Group on Sarcopenia in Older People 2 (EWGSOP2) consensus reflects this concept by recognizing low muscle strength as the primary indicator of probable sarcopenia, whereas reduced muscle quantity or quality confirms the diagnosis and impaired physical performance identifies severe sarcopenia [8]. Accordingly, assessment of handgrip strength, gait speed, chair-stand performance, and the Short Physical Performance Battery (SPPB) has become central to the clinical evaluation of sarcopenia [8]. Nevertheless, different operational definitions identify distinct sarcopenia phenotypes with different clinical implications [9]. Older adults with end-stage knee and hip OA constitute a particularly vulnerable population because prolonged pain, reduced mobility, chronic low-grade inflammation, and physical inactivity collectively accelerate skeletal muscle deterioration [10].

Chronic low-grade inflammation and oxidative stress have emerged as two interrelated biological mechanisms linking OA with skeletal muscle deterioration. Systemic inflammatory mediators, including C-reactive protein (CRP), have been associated with OA progression, impaired muscle function, and age-related declines in physical performance [11]. In parallel, excessive production of reactive oxygen species (ROS) promotes mitochondrial dysfunction, protein oxidation, lipid peroxidation, and activation of proteolytic pathways, thereby contributing to muscle atrophy and impaired muscle contractility [12,13,14,15]. Within the osteoarthritic joint, oxidative stress further accelerates cartilage degeneration through activation of inflammatory signaling pathways, extracellular matrix degradation, and cellular senescence [16,17,18]. Lipid peroxidation products, including F_2_-isoprostanes, are generated through free radical-mediated oxidation of arachidonic acid independently of cyclooxygenase activity and are considered among the most reliable biomarkers of in vivo oxidative stress [19,20]. Moreover, oxidative stress amplifies inflammatory signaling through activation of innate immune pathways and increased production of pro-inflammatory cytokines, thereby promoting cartilage degradation and progressive musculoskeletal dysfunction [21,22,23]. The neutrophil-to-lymphocyte ratio (NLR) has likewise emerged as a practical marker of systemic inflammation and immune dysregulation in OA [24], whereas activation of oxidative and inflammatory pathways within skeletal muscle accelerates protein degradation through the ubiquitin–proteasome system, thereby contributing to sarcopenia [25]. Collectively, these mechanisms may perpetuate a vicious cycle of chronic pain, functional decline, and progressive musculoskeletal deterioration in advanced OA [26].

Among the currently available biomarkers of oxidative stress, F_2_-isoprostanes are considered one of the most robust and reliable indicators of systemic lipid peroxidation [20]. Because they are chemically stable, generated in vivo, and readily measurable in urine after normalization to creatinine concentration, urinary F_2_-isoprostanes provide a non-invasive assessment of cumulative oxidative stress [20]. Elevated concentrations have been reported in synovial fluid, synovial tissue, serum, and urine of patients with OA, suggesting that oxidative stress reflects both local joint pathology and systemic biological alterations [27]. Furthermore, oxidative stress biomarkers have been associated with ageing, inflammation, and impaired skeletal muscle function in individuals with OA, supporting a potential relationship between redox imbalance and musculoskeletal decline [28].

Despite growing evidence linking OA, sarcopenia, inflammation and oxidative stress, their interrelationships in older adults with end-stage knee and hip OA remain incompletely understood. In particular, relatively few studies have simultaneously evaluated EWGSOP2-defined sarcopenia phenotypes together with muscle mass, muscle strength, physical performance, systemic inflammatory markers, and validated biomarkers of oxidative stress in patients undergoing primary knee or hip arthroplasty [29]. A better understanding of these relationships may facilitate identification of patients at increased risk of functional decline and support targeted preoperative rehabilitation, nutritional optimization, and individualized perioperative management. Addressing these knowledge gaps may also contribute to improved perioperative risk stratification and the development of interventions aimed at preserving muscle function and optimizing recovery following arthroplasty.

Although previous studies have investigated individual aspects of skeletal muscle impairment in osteoarthritis, including sarcopenia, systemic inflammation, oxidative stress, or physical performance, these factors have largely been examined independently [10,11,17,28]. Consequently, the complex interplay between muscle mass, muscle strength, physical performance, systemic inflammatory biomarkers, oxidative stress, and clinically relevant sarcopenia phenotypes remains incompletely characterized in older adults with end-stage knee and hip OA. In particular, studies simultaneously applying the updated EWGSOP2 diagnostic framework together with validated biomarkers of oxidative stress and inflammation to evaluate their independent associations with skeletal muscle health are scarce [28,29]. A more comprehensive understanding of these interrelationships may improve identification of patients at increased risk of functional decline and support the development of targeted preoperative and rehabilitation strategies. To our knowledge, few studies have examined these factors together in older adults with end-stage knee and hip osteoarthritis, particularly using the EWGSOP2 criteria alongside biomarkers of oxidative stress and systemic inflammation.

Accordingly, the present study aimed to investigate the associations between muscle mass, muscle strength, physical performance, systemic inflammation, and oxidative stress, and to characterize sarcopenia phenotypes according to the EWGSOP2 criteria in older adults with end-stage knee and hip osteoarthritis. Potential differences according to the affected joint (knee vs. hip OA) were additionally explored to determine whether the site of end-stage OA was associated with distinct muscle, functional, or biomarker profiles. We hypothesized that muscle impairment in end-stage OA would be characterized by deficits in both muscle strength and muscle mass, with muscle strength demonstrating stronger associations with physical performance, and that increased systemic inflammation and oxidative stress would be associated with poorer muscle status and physical function.

## 2. Materials and Methods

### 2.1. Study Design and Participants

This cross-sectional observational study included older adults with end-stage knee or hip OA who were scheduled to undergo primary total knee arthroplasty (TKA) or total hip arthroplasty (THA). Participants were consecutively recruited between August 2023 and December 2025 from the 1st Orthopedic Department of the 424 General Military Training Hospital of Thessaloniki, Greece.

The required sample size was estimated before participant recruitment. An a priori power analysis was performed using G*Power software (version 3.1.9.4) for an independent-samples t-test (two-tailed, α = 0.05, power = 0.80, allocation ratio = 1.0). A standardized effect size of Cohen’s d = 0.60 was assumed, representing a moderate-to-large between-group effect. This magnitude was considered clinically plausible based on previous evidence in patients with knee and hip OA reporting standardized effects of approximately 0.5–0.6 for clinically relevant outcomes [30,31].

The a priori analysis indicated that a total sample of 90 participants was required, with 45 participants planned for each of the knee OA and hip OA groups. Ultimately, 88 participants were enrolled (56 with knee OA and 32 with hip OA), reflecting the availability of eligible patients undergoing elective arthroplasty during the recruitment period. A sensitivity analysis based on the achieved group sizes (n = 56 and n = 32), α = 0.05, and power = 0.80 indicated that the final sample could detect a between-group effect of approximately Cohen’s d = 0.63 (Hedges’ g ≈ 0.62). Because the principal analyses also examined associations using hierarchical multiple linear regression, an additional sensitivity analysis was conducted for the most complex regression model. With N = 88, five predictors, α = 0.05, and power = 0.80, the minimum detectable effect was approximately f^2^ = 0.16, corresponding to a medium effect. Although the planned sample size was nearly achieved (88 of the required 90 participants), sensitivity analyses indicated that the study was adequately powered to detect moderate-to-large between-group differences and approximately medium multivariable associations. Consequently, smaller effects, particularly within the hip OA subgroup, may have remained undetected.

Eligible participants were adults with radiographically confirmed end-stage primary knee or hip OA who were scheduled to undergo elective primary TKA or THA. Exclusion criteria included the presence of implantable electronic devices, malignant disease, active autoimmune disorders, uncontrolled metabolic disease, dementia, neuromuscular disorders, severe hepatic or renal disease, acute inflammatory or infectious conditions, and any other medical condition that could substantially affect body composition, functional performance, cognitive status, or the inflammatory and oxidative stress biomarkers evaluated in the study.

All study assessments were performed during the routine preoperative evaluation before surgery using standardized protocols. Demographic characteristics, medical history, anthropometric measurements, body composition, muscle strength, physical performance, and biochemical markers of inflammation and oxidative stress were collected during the same preoperative visit.

The study was conducted in accordance with the ethical principles of the Declaration of Helsinki and was approved by the Scientific and Ethical Board of the 424 General Military Training Hospital of Thessaloniki (Approval No. 45/6th Session/24 July 2023/424 General Military Training Hospital/Education Sector Division).

Before enrolment, all participants received detailed verbal and written information regarding the study objectives and procedures. Written informed consent was obtained from all participants prior to participation. Participant confidentiality was ensured through coded data collection and anonymized data analysis in accordance with institutional and international ethical standards.

### 2.2. Anthropometric Measurements and Body Composition Assessment

Anthropometric measurements were performed according to standardized procedures. Body weight was measured to the nearest 0.1 kg using a calibrated digital scale, and standing height was measured to the nearest 0.1 cm using a wall-mounted stadiometer. Body mass index (BMI) was calculated as body weight divided by height squared (kg/m^2^) using a Tanita WB-380 scale (Tanita Corporation, Tokyo, Japan) and a built-in stadiometer, respectively—with subjects in light attire and no shoes to ensure an accurate BMI calculation.

Body composition was assessed by bioelectrical impedance analysis (BIA) using a BodyStat^®^ 1500 MDD analyzer (Bodystat Ltd., Douglas, Isle of Man, British Isles), which operates at dual frequencies of 5 and 50 kHz. All BIA measurements were performed by the same trained investigator according to standardized procedures. To minimize the influence of hydration status on impedance measurements, participants were assessed in the morning following an overnight fast, after bladder emptying, and were instructed to avoid vigorous physical activity and alcohol consumption during the preceding 24 h. Before the assessment, participants rested in the supine position for at least 5 min to allow body fluid stabilization. Surface electrodes were then placed according to the manufacturer’s guidelines, and measurements were obtained with participants remaining in the supine position.

The following body composition parameters were recorded: fat-free mass (FFM), fat mass (FM), body fat percentage (BF%), and appendicular skeletal muscle mass (ASMM). In particular, ASMM was estimated using multifrequency BIA according to the manufacturer’s prediction algorithm. Bioelectrical impedance-derived estimates of ASMM have previously been validated against dual-energy X-ray absorptiometry (DXA) in older adults and provide a valid estimate of appendicular skeletal muscle mass for sarcopenia assessment [31]. In accordance with the EWGSOP2 recommendations [8], muscle quantity was expressed as ASMM adjusted for height squared (ASMM/H^2^; kg/m^2^). In addition, ASMM adjusted for BMI (ASMM/BMI) was calculated as an alternative index of muscle mass.

Phase angle (PhA,°) was measured using multifrequency bioelectrical impedance analysis (BIA). The analyzer calculated phase angle at 50 kHz from the relationship between reactance (Xc) and resistance (R) according to the equation PhA = arctangent (Xc/R) × (180/π). Phase angle has been validated as a non-invasive indicator of cellular integrity and bioelectrical properties associated with skeletal muscle health [32,33].

### 2.3. Assessment of Sarcopenia According to the EWGSOP2 Criteria

Sarcopenia status was classified according to the updated EWGSOP2 consensus [29]. Probable sarcopenia was defined as low muscle strength, identified using sex-specific handgrip strength cut-offs (<27 kg for men and <16 kg for women). Confirmation of sarcopenia required the presence of low muscle quantity, defined as ASMM/H^2^ below the EWGSOP2 thresholds (<7.0 kg/m^2^ for men and <5.5 kg/m^2^ for women) [29]. Because only a limited number of participants fulfilled the criteria for confirmed sarcopenia, individuals with probable and confirmed sarcopenia were combined into a single category for statistical analyses.

Muscle strength was assessed by measuring maximal handgrip strength using a calibrated digital handgrip dynamometer (Takei Scientific Instruments Co., Ltd., Tokyo, Japan). Three maximal contractions were performed using the dominant hand with a 60-s rest interval between attempts, and the highest value was used for statistical analyses. Sex-specific EWGSOP2 cut-off values were applied to identify low muscle strength [29]. Participants who did not meet the criteria for low muscle strength were classified as non-sarcopenic.

Muscle quantity was assessed using ASMM/H^2^, as described in Section 2.2. Participants with low muscle strength were classified as having probable sarcopenia, whereas those with low muscle strength in combination with reduced muscle quantity were classified as having confirmed sarcopenia, according to the EWGSOP2 diagnostic criteria [29].

### 2.4. Assessment of Physical Performance

Lower-extremity physical performance was assessed using a standardized battery of objective functional tests, including gait speed, the five-times chair stand test, and the Short Physical Performance Battery (SPPB) [34]. All measurements were performed by the same trained investigator following standardized operating procedures to minimize inter-observer variability.

Gait speed was assessed over a 4-m walkway at each participant’s usual walking speed. Participants started from a standing position at the beginning of the walkway and were instructed to walk at their usual pace. Walking time was recorded manually using a stopwatch from the initiation of movement until the participant crossed the 4-m line. Two trials were performed, and the faster trial was used to calculate gait speed (m/s) for statistical analyses. Participants wore their usual footwear throughout the assessment and, when required, used their customary walking aids to ensure that the evaluation reflected their usual functional capacity. The SPPB yields a composite score ranging from 0 to 12, with lower scores indicating poorer physical performance [35]. Impaired physical performance was defined as an SPPB score ≤ 8 according to the EWGSOP2 recommendations [29,34,35].

The five-times chair stand test was also analyzed as an individual measure of lower-extremity muscle function. Participants were instructed to rise from a seated position and return to a seated position five consecutive times as quickly as possible without using their upper extremities for assistance. The time required to complete the test was recorded according to the standardized protocol described by Bohannon and colleagues [35,36].

Self-reported symptoms and disease-specific functional status were assessed using the Western Ontario and McMaster Universities Osteoarthritis Index (WOMAC). The WOMAC evaluates three domains—pain, stiffness, and physical function—in individuals with knee or hip osteoarthritis. Total WOMAC scores were calculated according to the validated scoring procedure [37].

### 2.5. Biochemical Analyses

Venous blood samples were collected during the routine preoperative evaluation following an overnight fast of at least 12 h. Serum C-reactive protein (CRP), complete blood count parameters, and biochemical markers of lipid metabolism and glycemic control, including total cholesterol, triglycerides, low-density lipoprotein cholesterol (LDL-C), high-density lipoprotein cholesterol (HDL-C), fasting plasma glucose, and glycated hemoglobin (HbA1c), were measured as part of the routine preoperative clinical evaluation. All biochemical analyses were performed at the Clinical Biochemistry Laboratory of the 424 General Military Training Hospital of Thessaloniki according to standardized laboratory procedures. The neutrophil-to-lymphocyte ratio (NLR) was calculated as the ratio of the absolute neutrophil count to the absolute lymphocyte count.

Participants receiving pharmacological treatment for dyslipidaemia, type 2 diabetes mellitus, or hypertension were considered to have the corresponding comorbidity, irrespective of the laboratory values measured at the time of study participation. Medication use at baseline was recorded from participants’ reports and cross-checked against their medical records. The medications considered were antidiabetic, lipid-lowering, antihypertensive, Non-Steroidal Anti-Inflammatory Drugs (NSAIDs), corticosteroid drugs. NSAIDs were not taken as regular treatment; according to the medical records, they were used as needed when pain occurred. Medication use was subsequently compared between participants with and without dyslipidaemia.

Oxidative stress was evaluated by measuring urinary F_2_-isoprostane concentrations. First-morning spot urine samples were collected during the preoperative evaluation and stored at −80 °C until analysis. Urinary F_2_-isoprostanes were quantified using a competitive enzyme-linked immunosorbent assay (ELISA) kit (Cayman Chemical, Ann Arbor, MI, USA) according to the manufacturer’s instructions. All urine samples were analyzed in duplicate, and assay precision was evaluated using intra- and inter-assay coefficients of variation, which were <4% and <7%, respectively. Urinary F_2_-isoprostane concentrations were normalized to urinary creatinine concentrations to account for variations in urine concentration. Normalized urinary F_2_-isoprostane concentrations were expressed as ng/mg creatinine and were used in all subsequent statistical analyses.

### 2.6. Statistical Analysis

All statistical analyses were performed using IBM SPSS Statistics version 23.0 (IBM Corp., Armonk, NY, USA). Continuous variables were assessed for normality using the Shapiro–Wilk test together with visual inspection of histograms and normal Q–Q plots. Variables exhibiting substantial positive skewness, including CRP, and urinary F_2_-isoprostanes, were natural log-transformed (ln) to satisfy the assumptions of parametric analyses. Normally distributed continuous variables are presented as mean ± standard deviation (SD), whereas categorical variables are presented as frequencies and percentages.

Between-group comparisons were performed using independent-samples *t*-tests for continuous variables. Homogeneity of variances was evaluated using Levene’s test, and Welch’s correction was applied when the assumption of equal variances was violated. Categorical variables were compared using Pearson’s chi-square test or Fisher’s exact test, as appropriate.

Participants were classified according to the EWGSOP2 diagnostic criteria as described in Section 2.3. Owing to the limited number of participants with confirmed or severe sarcopenia, these categories were combined with the probable sarcopenia group for inferential analyses. Pearson correlation coefficients were calculated using log-transformed values for non-normally distributed variables. Correlation coefficients were interpreted as weak (r = 0.10–0.29), moderate (r = 0.30–0.49), or strong (r ≥ 0.50). To account for multiple testing in the correlation analysis, the Benjamini–Hochberg false discovery rate (BH-FDR) procedure was applied across the 45 unique pairwise correlations, with an FDR-adjusted *p*-value < 0.05 considered statistically significant.

Multiple hierarchical linear regression analyses were performed according to predefined biological hypotheses. For analyses of physical performance, the SPPB score was entered as the dependent variable. Age and sex were entered in the first block, followed by muscle-related variables in subsequent blocks. A separate hierarchical regression model was constructed to identify independent predictors of ASMM/H^2^, with age and sex entered in the first block, followed by inflammatory biomarkers (ln-CRP and ln-NLR) and the oxidative stress biomarker (ln-urinary F_2_-isoprostanes).

Secondary exploratory analyses examined the associations between metabolic characteristics, dyslipidemia status, and ASMM/BMI. Categorical medication variables were compared between participants with and without dyslipidaemia using Fisher’s exact test because of the small cell counts in several medication categories. Percentages were calculated within each dyslipidaemia group. These analyses were considered exploratory and interpreted as hypothesis-generating.

Model performance was evaluated using the coefficient of determination (R^2^), adjusted R^2^, F-statistics, and changes in explained variance (ΔR^2^). Standardized regression coefficients (β), unstandardized regression coefficients (B), 95% confidence intervals (CI), and *p*-values are reported. The assumptions of linear regression (linearity, homoscedasticity, normality of residuals, independence, and multicollinearity) were verified before model interpretation. Multicollinearity was evaluated using variance inflation factors (VIF), with values <5 considered acceptable. No missing data were observed for the variables included in the statistical analyses.

The magnitude of between-group differences was quantified using Hedges’ g, with effect sizes interpreted as small (0.2), moderate (0.5), and large (≥0.8). The primary analyses were predefined according to the study objectives. No formal adjustment for multiple comparisons was applied to secondary exploratory analyses; therefore, these findings should be interpreted cautiously and regarded as hypothesis-generating rather than confirmatory. A two-sided *p*-value < 0.05 was considered statistically significant.

## 3. Results

### 3.1. Study Population and Baseline Characteristics

**Figure 1:**
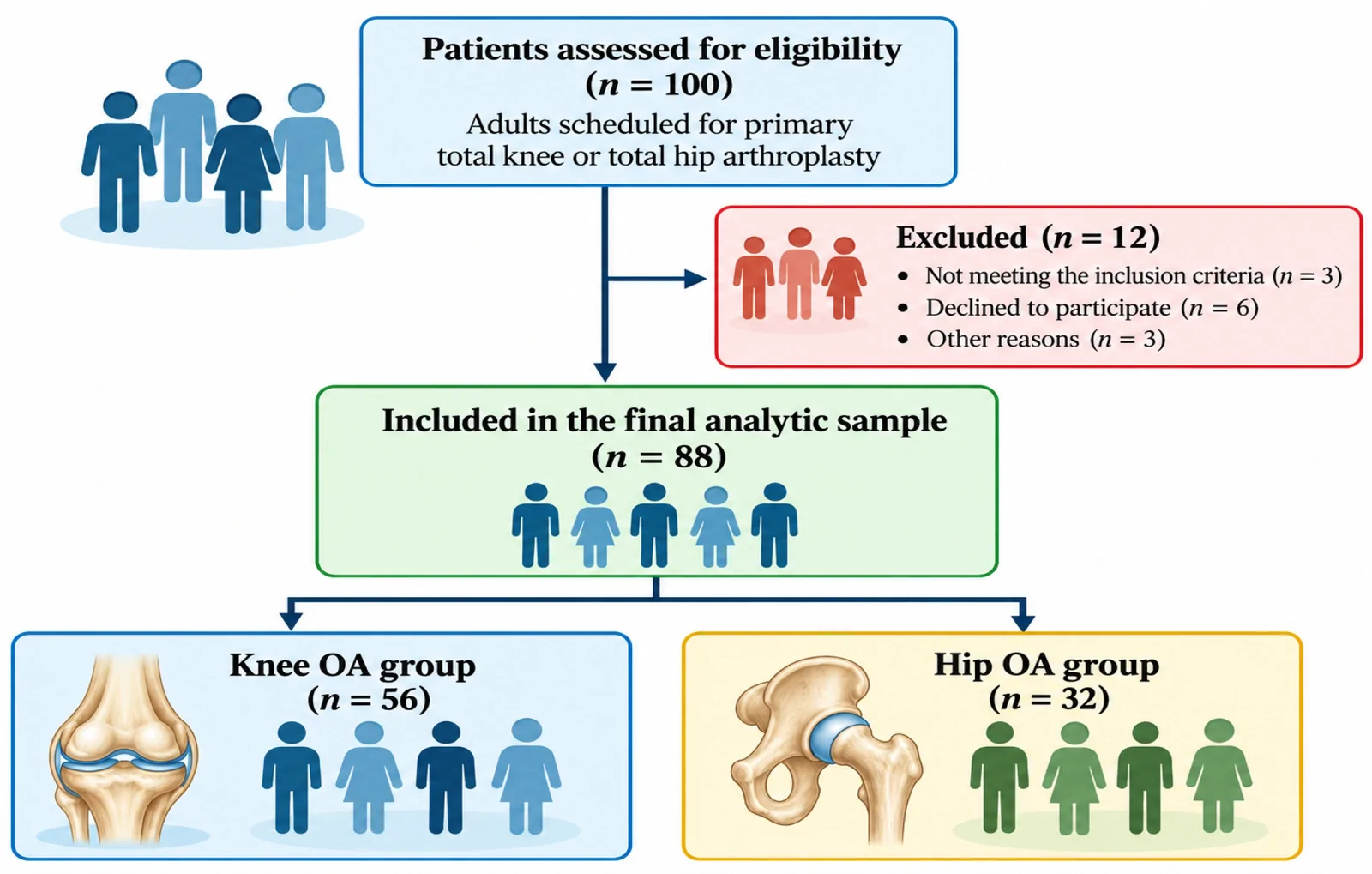
Flow diagram of participant screening, eligibility and inclusion in the final analytic sample.

A total of 88 older adults with end-stage OA scheduled for primary arthroplasty were included in the study, comprising 56 participants with knee OA and 32 participants with hip OA (Figure 1). Baseline body composition, physical performance, inflammatory, and oxidative stress characteristics according to the affected joint are presented in Table 1.

Overall, participants with knee and hip OA exhibited largely comparable body composition, inflammatory and oxidative stress biomarker profiles, and physical performance characteristics. No statistically significant between-group differences were observed in body composition, inflammatory or oxidative stress biomarkers, or physical performance measures, including lean mass, ASMM/H^2^, body water compartments, ln-transformed CRP, NLR, ln-transformed urinary F_2_-isoprostanes, SPPB score, chair-stand performance, or WOMAC scores.

Among all variables examined, phase angle was the only parameter that differed significantly between groups, with participants with knee OA exhibiting higher values than those with hip OA (5.70 ± 2.40° vs. 4.81 ± 0.71°; mean difference = 0.89°, 95% CI 0.19–1.58; *p* = 0.013; Hedges’ g = 0.45). Body fat percentage also tended to be lower in participants with knee OA than in those with hip OA (39.13 ± 10.46% vs. 43.22 ± 8.91%; mean difference = −4.09%, 95% CI −8.28 to 0.11; *p* = 0.056; Hedges’ g = −0.42). Body fat percentage also showed a trend to be lower in participants with knee OA than in those with hip OA (39.13 ± 10.46% vs. 43.22 ± 8.91%; mean difference = −4.09%, 95% CI −8.28 to 0.11; *p* = 0.056; Hedges’ g = −0.42).

### 3.2. EWGSOP2 Sarcopenia Phenotype Distribution

Using the EWGSOP2 diagnostic criteria (handgrip strength < 27/<16 kg and ASMM/H^2^ < 7.0/<5.5 kg/m^2^ for men/women), 26 participants (29.5%) were classified as having probable/confirmed sarcopenia (Table 2). Specifically, 22 participants (25.0%) were categorized as having probable sarcopenia based on low handgrip strength, while 4 participants (4.5%) fulfilled the criteria for confirmed sarcopenia, defined by the coexistence of low muscle strength and low ASMM/H^2^. All four participants with confirmed sarcopenia also had low physical performance (SPPB score ≤ 8) and therefore fulfilled the EWGSOP2 criteria for severe sarcopenia. Because of the small number of participants with confirmed/severe sarcopenia, these participants were combined with those with probable sarcopenia for subsequent inferential analyses. The distribution of probable/confirmed sarcopenia according to the affected joint is presented in Table 2. Probable/confirmed sarcopenia was identified in 17 of 56 participants (30.4%) with knee OA and 9 of 32 participants (28.1%) with hip OA, whereas 39 (69.6%) and 23 (71.9%) participants, respectively, were classified as non-sarcopenic. The distribution of sarcopenia phenotypes did not differ significantly between participants with knee and hip OA (χ^2^ = 0.049, *p* = 0.825).

### 3.3. Comparison of Body Composition, Muscle Strength, Physical Performance, and Biomarkers According to EWGSOP2 Sarcopenia Phenotype

As expected from the EWGSOP2 classification algorithm, participants categorized as having probable/confirmed sarcopenia exhibited significantly lower handgrip strength (11.36 ± 3.06 vs. 26.38 ± 11.93 kg; mean difference = 15.02 kg, 95% CI 10.45–19.59; *p* < 0.001; Hedges’ g = 1.49) and ASMM/H^2^ (7.86 ± 1.98 vs. 9.32 ± 2.32 kg/m^2^; mean difference = 1.46 kg/m^2^, 95% CI 0.47–2.45; *p* = 0.004; Hedges’ g = 0.65) than participants without sarcopenia. Because reduced muscle strength constitutes the criterion for probable sarcopenia and reduced muscle quantity contributes to confirmation of the diagnosis, these comparisons are presented primarily to characterize the phenotypic groups rather than as independent associations.

Beyond the variables incorporated into the EWGSOP2 classification, unadjusted analyses showed that participants with probable/confirmed sarcopenia had a significantly higher body fat percentage than those without sarcopenia (45.06 ± 6.41% vs. 38.75 ± 10.76%; mean difference = −6.31%, 95% CI −10.37 to −2.25; *p* = 0.001; Hedges’ g = 0.67) and lower gait speed (0.65 ± 0.26 vs. 0.87 ± 0.31 m/s; mean difference = 0.22 m/s, 95% CI 0.09–0.35; *p* = 0.001; Hedges’ g = 0.77). No significant differences were observed for ln(CRP) (*p* = 0.550) or NLR (*p* = 0.683). ln(Urinary F_2_-isoprostanes) were higher in the probable/confirmed sarcopenia group, although the difference did not reach statistical significance (*p* = 0.053; Hedges’ g = 0.48) (Table 3).

To account for the observed age difference between phenotype groups, an additional age-adjusted multivariate analysis was performed for variables not incorporated into the EWGSOP2 classification algorithm. After adjustment for age, the previously observed differences in body fat percentage and gait speed were attenuated and were no longer statistically significant (*p* = 0.126 and *p* = 0.140, respectively). No significant age-adjusted differences were observed for ln(CRP) (*p* = 0.295), NLR (*p* = 0.606), or ln(urinary F_2_-isoprostanes) (*p* = 0.092). The overall multivariate effect of sarcopenia phenotype was also not statistically significant (Pillai’s Trace = 0.100, F(5,80) = 1.782, *p* = 0.126, partial η^2^ = 0.100) (Table 4).

### 3.4. Associations Between Muscle-Related Parameters, Physical Performance, and Biomarkers

**Figure 2:**
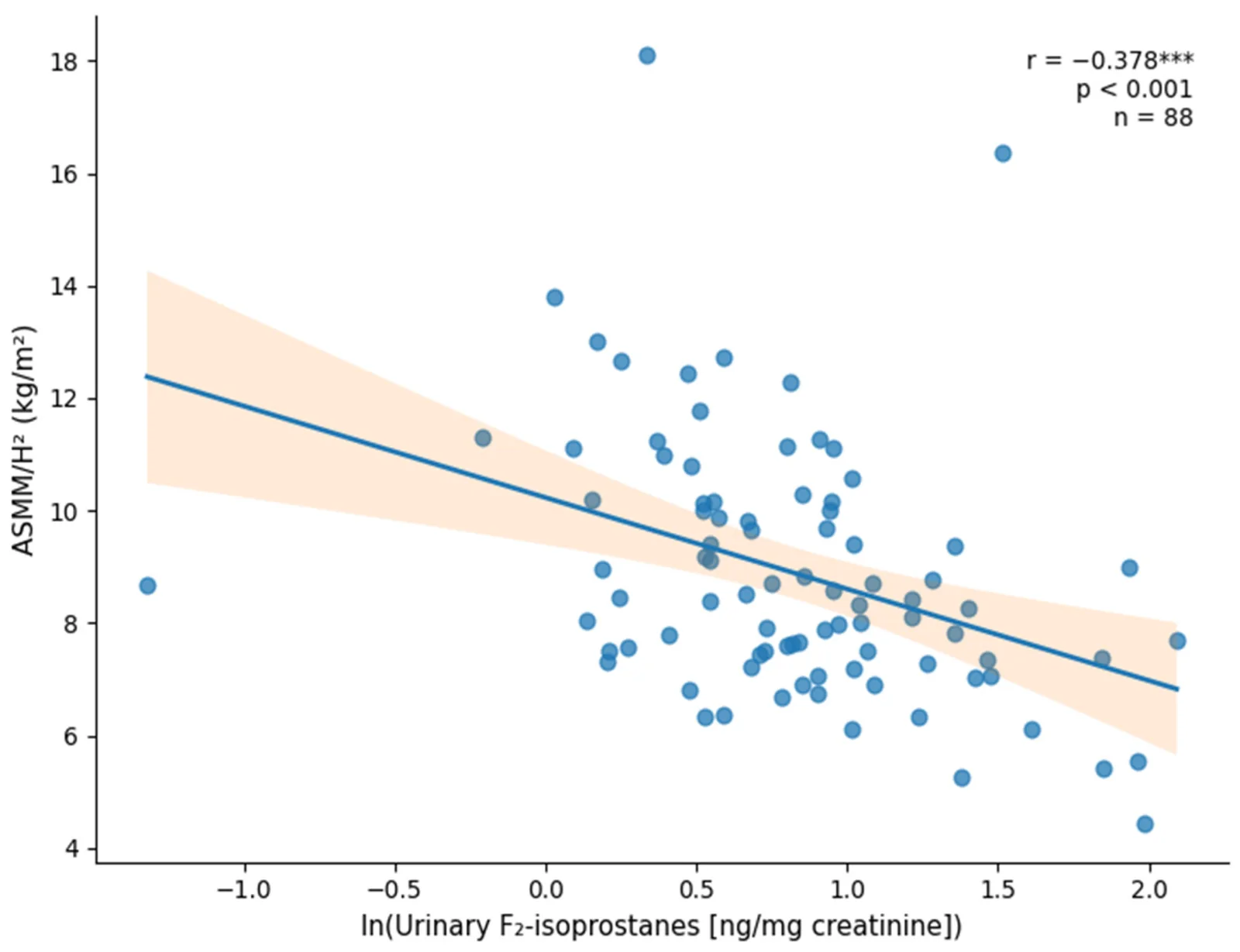
Relationship between urinary F_2_-isoprostanes and appendicular skeletal muscle mass adjusted for height squared (ASMM/H^2^) in older adults with end-stage knee and hip osteoarthritis. Urinary F_2_-isoprostane concentrations were natural logarithm-transformed before analysis. The solid line represents the fitted linear regression line. The shaded area represents the 95% confidence interval. *** indicates *p* < 0.001.

Pearson correlation analyses were performed to examine the associations among muscle-related parameters, physical performance, inflammatory biomarkers, and oxidative stress biomarkers (Table 5). ASMM/H^2^ demonstrated a strong positive correlation with ASMM/BMI (r = 0.738, *p* < 0.001) and was also strongly correlated with handgrip strength (r = 0.516, *p* < 0.001). Conversely, ASMM/H^2^ was moderately and inversely associated with ln-transformed urinary F_2_-isoprostanes (r = −0.378, *p* < 0.001; Figure 2) and showed weak inverse correlations with ln-transformed CRP (r = −0.246, *p* = 0.021) and NLR (r = −0.232, *p* = 0.029). These associations remained statistically significant after Benjamini–Hochberg False Discovery Rate (BH-FDR) correction.

Muscle strength was strongly associated with physical performance. Handgrip strength demonstrated strong positive correlations with gait speed (r = 0.635, *p* < 0.001) and the SPPB score (r = 0.539, *p* < 0.001), indicating that greater muscle strength was associated with better lower-extremity function. Similarly, ASMM/BMI was positively correlated with gait speed (r = 0.475, *p* < 0.001) and SPPB score (r = 0.446, *p* < 0.001). All these associations remained significant after BH-FDR correction.

Phase angle was positively associated with indices of muscle quantity and function, correlating with ASMM/H^2^ (r = 0.448, *p* < 0.001), ASMM/BMI (r = 0.473, *p* < 0.001), handgrip strength (r = 0.491, *p* < 0.001), gait speed (r = 0.417, *p* < 0.001), and SPPB score (r = 0.452, *p* < 0.001). Conversely, the five-times chair stand test demonstrated moderate-to-strong inverse correlations with ASMM/H^2^ (r = −0.378, *p* < 0.001), ASMM/BMI (r = −0.462, *p* < 0.001), handgrip strength (r = −0.623, *p* < 0.001), gait speed (r = −0.684, *p* < 0.001), and SPPB score (r = −0.701, *p* < 0.001), indicating poorer lower-extremity performance among participants with lower muscle mass and muscle strength. These associations remained significant after BH-FDR correction.

Regarding the lipid peroxidation biomarker, F2-isoprostanes in urine, ln-transformed urinary F_2_-isoprostanes demonstrated moderate inverse associations with ASMM/BMI (r = −0.332, *p* = 0.002), handgrip strength (r = −0.391, *p* < 0.001), gait speed (r = −0.351, *p* = 0.001), SPPB score (r = −0.298, *p* = 0.004), and phase angle (r = −0.305, *p* = 0.004), suggesting that higher oxidative stress was associated with lower muscle mass, reduced muscle strength, poorer physical performance, and lower phase angle. All these associations remained statistically significant after BH-FDR correction.

Inflammatory biomarkers demonstrated comparatively weaker associations with muscle-related outcomes. ln-transformed CRP showed a weak inverse correlation with ASMM/H^2^ (r = −0.246, *p* = 0.021), which remained significant after BH-FDR correction. The inverse correlations of ln-transformed CRP with handgrip strength (r = −0.214, *p* = 0.045) and gait speed (r = −0.223, *p* = 0.037) did not remain statistically significant after BH-FDR correction. NLR was weakly inversely associated with ASMM/H^2^ (r = −0.232, *p* = 0.029), and this association remained significant after BH-FDR correction. ln-transformed CRP and NLR were positively correlated (r = 0.391, *p* < 0.001), an association that also remained significant after correction.

Overall, these findings indicate that the associations of higher oxidative stress with lower muscle mass, reduced muscle strength, poorer physical performance, and lower phase angle were robust to BH-FDR correction, whereas associations involving systemic inflammatory biomarkers were comparatively weaker and less consistent after correction for multiple testing. Complete Pearson correlation coefficients and BH-FDR-adjusted significance levels are presented in Table 5.

### 3.5. Factors Associated with Physical Performance

Multiple hierarchical linear regression analyses were performed to identify independent factors associated with physical performance, assessed by the SPPB score (Table 6). In the initial hierarchical model, age and sex were entered as demographic covariates. The model was statistically significant (R^2^ = 0.129; adjusted R^2^ = 0.108; F = 6.139; *p* = 0.003) and explained 12.9% of the variance in SPPB score. Increasing age was independently associated with lower SPPB performance (B = −0.070, β = −0.348, 95% CI (−0.112, −0.029), *p* = 0.001), whereas sex was not significantly associated with SPPB score (*p* = 0.938).

To determine whether muscle-related variables contributed to physical performance beyond demographic characteristics, handgrip strength and ASMM/H^2^ were entered in the second hierarchical model. The inclusion of these variables significantly improved model performance, increasing the explained variance from 12.9% to 21.3% (ΔR^2^ = 0.084; adjusted R^2^ = 0.174; F = 4.338; *p* = 0.002). After adjustment for age and sex, handgrip strength remained independently associated with better physical performance (B = 0.050, β = 0.264, 95% CI (0.003, 0.097), *p* = 0.039), whereas ASMM/H^2^ was not independently associated with SPPB score (B = 0.074, β = 0.074, 95% CI (−0.153, 0.301), *p* = 0.520). Age also remained an independent predictor of lower SPPB scores (B = −0.047, β = −0.235, 95% CI (−0.090, −0.002), *p* = 0.039), while sex remained non-significant (*p* = 0.290).

Overall, the final hierarchical model demonstrated that muscle strength, but not muscle quantity, independently contributed to physical performance after adjustment for age and sex. Specifically, the addition of muscle-related variables increased the explained variance in SPPB score by 8.4%, although only handgrip strength remained an independent predictor in the fully adjusted model. These findings indicate that handgrip strength remained independently associated with physical performance after adjustment for age, sex, and ASMM/H^2^, whereas ASMM/H^2^ was not independently associated with SPPB score in the same model. Notably, the addition of handgrip strength and ASMM/H^2^ increased the explained variance in SPPB score by 8.4% (ΔR^2^ = 0.084). Handgrip strength remained independently associated with physical performance after adjustment for age, sex, and ASMM/H^2^ (β = 0.264, *p* = 0.039), whereas ASMM/H^2^ was not independently associated with SPPB score in the same model (β = 0.074, *p* = 0.520), (Table 6).

### 3.6. Factors Associated with Appendicular Skeletal Muscle Mass Index (ASMM/H^2^)

Multiple hierarchical linear regression analyses were performed to identify independent factors associated with ASMM/H^2^ (Table 7 ). In the initial hierarchical model, age and sex were entered as demographic covariates. The model was statistically significant (R^2^ = 0.172; adjusted R^2^ = 0.152; *p* < 0.001) and explained 17.2% of the variance in ASMM/H^2^. Sex was independently associated with ASMM/H^2^, with men demonstrating significantly higher muscle mass than women (B = 1.946, β = 0.391, 95% CI (0.946, 2.945), *p* < 0.001), whereas age was not significantly associated with ASMM/H^2^ (*p* = 0.427).

To determine whether inflammatory and oxidative stress biomarkers contributed to muscle mass beyond demographic characteristics, ln-transformed CRP and ln-transformed urinary F_2_-isoprostanes were entered in the second hierarchical model. The addition of these biomarkers significantly improved model performance, increasing the explained variance from 17.2% to 29.5% (ΔR^2^ = 0.123; adjusted R^2^ = 0.261; *p* < 0.001). In this model, both ln-transformed CRP and ln-transformed urinary F_2_-isoprostanes were significantly associated with ASMM/H^2^, while sex remained an independent predictor and age remained non-significant.

In the final hierarchical model, NLR was additionally included to evaluate whether oxidative stress remained independently associated with muscle mass after adjustment for multiple inflammatory biomarkers. The final model explained 29.8% of the variance in ASMM/H^2^ (R^2^ = 0.298; adjusted R^2^ = 0.255; *p* < 0.001). After full adjustment, ln-transformed urinary F_2_-isoprostanes remained independently associated with lower ASMM/H^2^ (B = −1.147, β = −0.265, 95% CI (−1.991, −0.303), *p* = 0.008), whereas ln-transformed CRP and NLR were no longer statistically significant. Sex also remained independently associated with ASMM/H^2^, while age continued to show no significant association.

Overall, the hierarchical regression analysis demonstrated that oxidative stress, represented by ln-transformed urinary F_2_-isoprostanes, was independently associated with lower appendicular skeletal muscle mass after adjustment for demographic characteristics and inflammatory biomarkers. In contrast, ln-transformed CRP and NLR did not retain independent associations in the fully adjusted model, suggesting that oxidative stress may represent a stronger correlate of reduced muscle mass than systemic inflammation in older adults with end-stage knee and hip OA.

### 3.7. Factors Associated with Relative Muscle Mass (ASMM/BMI)

Multiple hierarchical linear regression analyses were performed to identify independent factors associated with ASMM/BMI (Table 8). In the initial hierarchical model, age and sex were entered as demographic covariates. The model was statistically significant (R^2^ = 0.381; adjusted R^2^ = 0.366; F = 26.159; *p* < 0.001) and explained 38.1% of the variance in ASMM/BMI. Increasing age was independently associated with lower ASMM/BMI (B = −0.008, β = −0.402, 95% CI (−0.012, −0.005), *p* < 0.001), whereas sex was independently associated with ASMM/BMI, with men demonstrating significantly higher relative muscle mass than women (B = 0.211, β = 0.338, 95% CI (0.113, 0.309), *p* < 0.001).

To determine whether urinary F_2_-isoprostanes were associated with relative muscle mass beyond demographic characteristics, ln-transformed urinary F_2_-isoprostanes were added in the second hierarchical model. The inclusion of ln-transformed urinary F_2_-isoprostanes increased the explained variance from 38.1% to 41.4% (ΔR^2^ = 0.033; adjusted R^2^ = 0.393; F = 19.782; *p* < 0.001). After adjustment for age and sex, higher ln-transformed urinary F_2_-isoprostane concentrations were independently associated with lower ASMM/BMI (B = −0.082, β = −0.188, 95% CI (−0.158, −0.007), *p* = 0.034), while both age and sex remained significant independent predictors.

In the final hierarchical model, NLR was additionally entered to examine whether the association between urinary F_2_-isoprostanes and relative muscle mass persisted after accounting for systemic inflammation. The final model was statistically significant (R^2^ = 0.430; adjusted R^2^ = 0.402; F = 15.654; *p* < 0.001) and explained 43.0% of the variance in ASMM/BMI. The inclusion of NLR resulted in a further increase in explained variance (ΔR^2^ = 0.016). After full adjustment, age (B = −0.008, β = −0.392, 95% CI (−0.012, −0.004), *p* < 0.001) and sex (B = 0.203, β = 0.324, 95% CI (0.104, 0.302), *p* < 0.001) remained independently associated with ASMM/BMI. The association with ln-transformed urinary F_2_-isoprostanes was attenuated and did not reach statistical significance (B = −0.076, β = −0.174, 95% CI (−0.155, 0.004), *p* = 0.060), while NLR was also not significantly associated with ASMM/BMI (B = −0.027, β = −0.131, 95% CI (−0.063, 0.009), *p* = 0.136).

Taken together, the hierarchical regression analysis identified age and gender as independent predictors of relative muscle mass (ASMM/BMI) in older adults with end-stage OA. Higher ln-transformed urinary F_2_-isoprostane concentrations were independently associated with lower ASMM/BMI after adjustment for age and sex and contributed an additional 3.3% to the explained variance (ΔR^2^ = 0.033). Following the addition of NLR, this association was attenuated and did not reach statistical significance (β = −0.174, *p* = 0.060), while the final model explained 43.0% of the variance in ASMM/BMI.

### 3.8. Exploratory Analysis According to Dyslipidemia Status

In this secondary exploratory analysis, medication use in the group with and without dyslipidaemia is shown in Table 9**.** Lipid-lowering medication was used by all participants with dyslipidaemia (32/32, 100.0%) and by none of those without dyslipidaemia (0/56, 0.0%; Fisher’s exact test, *p* < 0.001). Antihypertensive medication was used by 30 participants without dyslipidaemia (53.6%) and 15 participants with dyslipidaemia (46.9%), with no significant difference between the groups (*p* = 0.658). Antidiabetic medication was reported in 8 participants without dyslipidaemia (14.3%) and 2 with dyslipidaemia (6.3%; *p* = 0.316). NSAID use for pain relief was reported in 42 (75.0%) and 27 (84.4%) participants, respectively (*p* = 0.421). Only one participant in the no-dyslipidaemia group was receiving corticosteroids (1.8%), while none of the participants with dyslipidaemia were receiving this medication (*p* = 1.000).

Participants were stratified according to dyslipidemia status to examine potential differences in body composition, muscle-related parameters, inflammatory biomarkers, and oxidative stress (Table 10). Individuals with dyslipidemia exhibited significantly higher body fat percentage than those without dyslipidemia (44.15 ± 6.88% vs. 38.62 ± 11.11%; mean difference = −5.53%, 95% CI (−9.83, −1.23); *p* = 0.014; Hedges’ g = 0.55). They also demonstrated significantly lower ASMM/BMI, indicating lower relative muscle mass (0.66 ± 0.18 vs. 0.76 ± 0.25; mean difference = 0.10, 95% CI (0.003, 0.189); *p* = 0.044; Hedges’ g = 0.43).

A significant difference was also observed in ln-transformed urinary F_2_-isoprostane concentrations, with lower concentrations among participants with dyslipidemia than among those without dyslipidemia (0.67 ± 0.53 vs. 0.90 ± 0.53; mean difference = 0.23, 95% CI (0.001, 0.459); *p* = 0.049; Hedges’ g = 0.43).

No statistically significant differences were observed between participants with and without dyslipidemia in ASMM/H^2^, lean mass, phase angle, physical performance (SPPB score), inflammatory biomarkers (ln-transformed CRP and NLR), or disease-specific functional status assessed by the WOMAC total score. Nevertheless, participants with dyslipidemia tended to report higher WOMAC scores than those without dyslipidemia (65.70 ± 14.95% vs. 59.13 ± 14.93%; mean difference = −6.57%, 95% CI (−13.25, 0.11); *p* = 0.053; Hedges’ g = 0.44).

Taken together, these exploratory findings showed that participants with dyslipidemia had greater adiposity and lower relative muscle mass than those without dyslipidemia. Lower ln-transformed urinary F_2_-isoprostane concentrations were also observed among participants with dyslipidemia; however, this unexpected finding should be interpreted cautiously because it emerged from a secondary exploratory analysis and requires confirmation in larger prospective studies. No statistically significant differences were observed in systemic inflammatory biomarkers or physical performance measures.

## 4. Discussion

The present study investigated sarcopenia phenotypes according to the updated EWGSOP2 criteria and examined their associations with muscle mass, muscle strength, physical performance, inflammatory biomarkers, and oxidative stress in older adults with end-stage knee and hip osteoarthritis. Nearly one third (29.5%) of the participants were classified as having probable/confirmed sarcopenia, highlighting the substantial burden of skeletal muscle impairment in this population. As expected according to the EWGSOP2 diagnostic algorithm, participants with probable/confirmed sarcopenia exhibited lower handgrip strength and ASMM/H^2^ than non-sarcopenic participants, since reduced muscle strength constitutes the primary criterion for probable sarcopenia and reduced muscle quantity is required for confirmation of the diagnosis [29]. Accordingly, these differences should primarily be interpreted as descriptive characteristics of the EWGSOP2 phenotypes rather than as independent associations. Beyond these expected differences, handgrip strength remained independently associated with physical performance in the multivariable analysis, while higher urinary F_2_-isoprostane concentrations were associated with poorer muscle characteristics. By examining these factors together, the present study provides a more integrated view of skeletal muscle impairment in older adults with end-stage knee and hip osteoarthritis.

The proportion of participants classified as having probable or confirmed sarcopenia (29.5%) in the present study is consistent with previous reports indicating that skeletal muscle impairment frequently accompanies advanced osteoarthritis, although estimates vary according to the study population, disease severity, and diagnostic criteria applied [10,11,36]. According to the EWGSOP2 consensus, low muscle strength is the primary criterion for identifying probable sarcopenia, whereas reduced muscle quantity or quality is required for confirmation [29]. Although the lower handgrip strength observed in the probable/confirmed sarcopenia group is expected because it forms part of the diagnostic algorithm, muscle strength remains clinically important because it is more closely associated with mobility limitation, disability, and adverse health outcomes than muscle mass alone [7].

Consistent with this concept, handgrip strength demonstrated stronger associations with physical performance than ASMM/H^2^ across the entire study population and remained independently associated with SPPB score after adjustment for age, sex, and muscle mass. In contrast, ASMM/H^2^ was not independently associated with physical performance in the fully adjusted regression model. These findings are in line with evidence showing that muscle strength may be more closely related to function than muscle quantity alone. They also suggest that a dynapenic component may be present in the muscle impairment observed in advanced OA [5]. Previous longitudinal studies have similarly demonstrated that declines in muscle strength and muscle quality contribute substantially to mobility limitation and functional deterioration, even when reductions in muscle mass are relatively modest [6,7]. Collectively, these findings underline the relevance of muscle strength and physical performance when examining skeletal muscle health in older adults with end-stage osteoarthritis.

The comparisons between knee and hip OA were exploratory. Sarcopenia phenotype distribution did not differ between the two groups, although phase angle was lower in participants with hip OA. Phase angle has been associated with cellular integrity, muscle strength, physical performance, and nutritional status in older adults [34], but it is also affected by hydration, nutritional status, and body composition and cannot be regarded as a direct measure of muscle quality. Muscle weakness, impaired endurance, neuromuscular dysfunction, and reduced muscle quality have been described in OA [4] and previous studies and systematic reviews have reported deficits in muscle strength and endurance [4,28]. Our finding of a similar sarcopenia phenotype distribution in knee and hip OA is broadly consistent with evidence that skeletal muscle impairment occurs in both conditions [10]. These exploratory comparisons, however, need confirmation in larger samples. Moreover, the absence of a statistically significant difference in sarcopenia phenotype distribution should not be interpreted as evidence of equivalence between the knee and hip OA groups, particularly given the unequal group sizes.

One of the most clinically relevant results of the present study is the independent association between urinary F_2_-isoprostanes and ASMM/H^2^. Specifically, higher urinary F_2_-isoprostane concentrations were independently associated with lower skeletal muscle mass after adjustment for age, sex, and inflammatory biomarkers, showing that greater oxidative stress was associated with lower skeletal muscle mass in older adults with end-stage OA. These findings are consistent with recent systematic reviews reporting significant associations between oxidative stress biomarkers and impaired muscle function in patients with OA [28]. Biologically, this relationship is plausible because excessive reactive oxygen species (ROS) production promotes mitochondrial dysfunction, impairs protein synthesis, activates proteolytic pathways, disrupts satellite cell activity, and accelerates muscle atrophy and regeneration failure [12,13,14,15,38]. Oxidative stress has also been implicated in synovial inflammation, cartilage degeneration, and cellular senescence, supporting the concept that common redox-dependent mechanisms may simultaneously contribute to OA progression and skeletal muscle deterioration [17,18]. Notably, the association between urinary F_2_-isoprostanes and ASMM/H^2^ remained significant after adjustment for CRP and NLR, suggesting that oxidative stress biomarkers may provide complementary information beyond conventional systemic inflammatory biomarkers when evaluating skeletal muscle health in advanced OA. Nevertheless, this finding should be interpreted cautiously because only one oxidative stress biomarker and two relatively nonspecific inflammatory biomarkers were evaluated, and the cross-sectional design precludes conclusions regarding causality. Future longitudinal and interventional studies should determine whether oxidative stress represents merely a marker of disease severity or a modifiable therapeutic target. Whether targeting oxidative stress or muscle health can influence outcomes before or after arthroplasty remains to be examined in prospective and interventional studies.

Interestingly, inflammatory biomarkers alone were not independently associated with ASMM/H^2^ or physical performance in the fully adjusted regression models. Although chronic low-grade inflammation is considered a central contributor to the pathogenesis of sarcopenia, evidence from systematic reviews indicates that associations between circulating inflammatory biomarkers and muscle strength or muscle mass are generally modest and heterogeneous [11,38]. Consequently, systemic inflammatory biomarkers such as CRP and the NLR may not fully capture the local biological processes occurring within skeletal muscle tissue. This may partly account for the relatively weak associations observed with the inflammatory markers in our study [38]. In this context, although NLR has emerged as a practical systemic inflammatory marker in OA, its relationship with skeletal muscle impairment remains less well established [24]. Our findings suggest that urinary F_2_-isoprostanes may provide complementary information regarding skeletal muscle impairment beyond that obtained from the conventional systemic inflammatory biomarkers evaluated in the present study. However, because only one oxidative stress biomarker and two relatively nonspecific inflammatory biomarkers (CRP and NLR) were examined, these findings should be interpreted cautiously and require confirmation using a broader panel of oxidative stress and inflammatory biomarkers in prospective mechanistic studies. Taken together, these findings suggest that the muscle changes seen in advanced OA are unlikely to be explained by a single biological pathway and may involve biomechanical, inflammatory, metabolic, and oxidative processes.

The findings for ASMM/BMI were secondary but may also be relevant when adiposity is considered. Relative muscle mass can provide information that differs from height-adjusted muscle mass, particularly in individuals with excess adiposity. This is consistent with the concept of sarcopenic obesity, in which reduced muscle reserve coexists with increased adiposity and has been associated with impaired mobility, metabolic dysfunction, poorer health outcomes, and increased disability [39]. In the present study, however, ASMM/BMI should be viewed as a complementary measure rather than a primary finding.

A further exploratory outcome was the lower urinary F_2_-isoprostane concentration among participants with dyslipidemia. This was unexpected, as dyslipidemia is generally associated with greater oxidative stress. Lipid-lowering treatment, particularly statin use, may be one possible explanation, since statins have antioxidant and anti-inflammatory effects. However, this analysis was exploratory and the difference was of borderline statistical significance. It should therefore be interpreted cautiously and examined further in studies specifically designed to assess the potential effects of lipid-lowering medication.

The strong associations observed between muscle-related parameters and physical performance further emphasize the close relationship between muscle characteristics and standardized performance-based assessments in older adults with advanced OA. Objective measures such as gait speed, the five-times chair stand test, and the SPPB provide complementary information on lower-extremity function and have consistently been associated with disability and other adverse health outcomes in older populations [40,41,42]. Collectively, these findings show that muscle strength and physical performance measures provide related but complementary information on functional status in patients awaiting arthroplasty.

From a clinical perspective, the observed associations draw attention to several aspects of skeletal muscle health in older adults with advanced OA, particularly muscle strength, physical performance, and body composition. The associations observed with oxidative stress may also be relevant, although their clinical significance remains uncertain. Given the cross-sectional design, the present study cannot determine whether routine assessment of these measures would improve clinical decision-making or whether resistance exercise, nutritional optimization, or structured prehabilitation would improve outcomes. These questions require prospective and interventional investigation.

The present cohort has several strengths. It provides a comprehensive assessment of skeletal muscle health in a clinically well-defined population of older adults with end-stage knee and hip OA, combining measures of muscle mass and strength with standardized physical performance tests and inflammatory and lipid peroxidation biomarkers. Sarcopenia phenotypes were classified according to the EWGSOP2 criteria, while the analytical approach included both correlation and multivariable regression analyses to examine the relationships among these parameters.

Several limitations should be acknowledged. First, participants were recruited from a single tertiary orthopedic center, which may limit the generalizability of the findings to other populations. Second, although the planned sample size was nearly achieved (88 of the required 90 participants), the study was primarily powered to detect medium-to-large effect sizes. Consequently, smaller associations, particularly within the hip OA subgroup, may have remained undetected. Third, because only a limited number of participants fulfilled the criteria for confirmed sarcopenia, probable and confirmed sarcopenia were combined for inferential analyses, precluding comparisons across individual sarcopenia stages. Fourth, residual confounding from unmeasured variables such as nutritional status and habitual physical activity cannot be excluded. Although medication use was examined in the exploratory analysis according to dyslipidemia status, medication variables were not systematically incorporated as covariates into the multivariable regression models. In addition, oxidative stress was assessed using urinary F_2_-isoprostanes as a marker of lipid peroxidation; therefore, the findings cannot be generalized to overall redox status. The inflammatory assessment was also limited to CRP and NLR. Other inflammatory cytokines, myokines, and antioxidant enzymes potentially involved in skeletal muscle homeostasis were not measured and therefore could not be evaluated.

The Benjamini–Hochberg false discovery rate correction was applied to the correlation analysis to account for multiple testing. The exploratory knee-versus-hip and dyslipidemia-related comparisons were not formally adjusted for multiple comparisons and should therefore be interpreted with caution. Finally, the cross-sectional design does not permit conclusions regarding causality or the temporal direction of the observed associations between urinary F_2_-isoprostanes and muscle-related outcomes. As the study was designed to investigate associations within a clinically defined population with end-stage OA, no OA-free comparison group was included. Consequently, the observed muscle mass, inflammatory, and oxidative stress characteristics should be interpreted within this specific clinical population and cannot be attributed specifically to OA.

Although our study included only patients with end-stage OA, some of the observed patterns may also be relevant to earlier stages of the disease. Previous longitudinal evidence in early knee OA has shown that changes in muscle strength are associated with changes in physical limitations over time [43]. Muscle strength, physical performance, and body composition may therefore be useful parameters to examine in future studies of sarcopenia in patients with earlier-stage OA. Elevated F_2_-isoprostane levels have also been reported in arthritic diseases, including OA [44]. Urinary F_2_-isoprostanes may be of particular interest in this context as a non-invasive marker of lipid peroxidation, although evidence specifically linking urinary F_2_-isoprostanes to early OA or to the risk of sarcopenia remains limited. Since patients with early OA were not included in our study, these findings cannot be directly extended to this population. Longitudinal studies are needed to determine whether these factors are associated with the development of sarcopenia as OA progresses.

In summary, the present findings indicate that skeletal muscle impairment in advanced knee and hip OA may be characterized predominantly by deficits in muscle strength and physical performance, whereas higher urinary F_2_-isoprostane concentrations were associated with several measures of poorer muscle status. Collectively, these observations highlight the close relationships between muscle characteristics, physical function and urinary F_2_-isoprostanes in end-stage OA. These associations are biologically plausible within the inflammatory and oxidative environment associated with advanced OA, although the cross-sectional design does not establish their temporal or causal direction. Longitudinal and interventional studies are needed to clarify these relationships and their clinical relevance.

## 5. Conclusions

The results of the present study highlight the close association between muscle strength, physical performance, body composition and urinary F2-isoprostanes in older adults with end-stage knee and hip OA. Furthermore, urinary F_2_-isoprostanes concentration was independently associated with lower skeletal muscle mass beyond conventional inflammatory biomarkers. Given the cross-sectional design, these findings do not establish causality or the clinical benefit of specific interventions.

Future longitudinal studies are needed to clarify the temporal associations between urinary F_2_-isoprostanes and muscle characteristics, while interventional studies are needed to determine whether strategies aimed at preserving muscle health can improve outcomes in patients undergoing total knee or hip arthroplasty.

## Figures and Tables

**Table 1:** Body composition, physical performance, inflammatory, and oxidative stress characteristics of participants according to the affected joint.

Variable	Knee OA (*n* = 56) Mean ± SD/IQR	Hip OA (*n* = 32) Mean ± SD/IQR	Mean Difference (95% CI)	*p*-Value	Hedges’ g
Age (years)	71.5 ± 6.2	71.3 ± 6.1	0.20 (−2.02, 1.71)	0.884	+0.03
Gender (male/female)	25/31	14/18	—	0.873	—
Body composition					
BMI (kg/m^2^)	27.9 ± 4.0	27.6 ± 4.2	0.30 (−1.53, 2.13)	0.744	+0.07
Lean mass (kg)	50.99 ± 15.60	46.54 ± 9.45	4.45 (−0.90, 9.79)	0.102	+0.35
Body fat (%)	39.13 ± 10.46	43.22 ± 8.91	−4.09 (−8.28, 0.11)	0.056	−0.42
ASMM/H^2^ (kg/m^2^)	8.9 ± 2.4	8.8 ± 2.3	0.04 (−0.99, 1.07)	0.939	0.02
Total body water (L)	40.13 ± 8.69	40.41 ± 8.04	−0.28 (−4.01, 3.45)	0.881	−0.03
Extracellular water (L)	7.50 ± 5.13	6.47 ± 6.74	1.03 (−1.51, 3.57)	0.421	+0.18
Intracellular water (L)	19.06 ± 4.54	19.30 ± 4.54	−0.24 (−2.24, 1.76)	0.811	−0.05
Phase angle (°)	5.70 ± 2.40	4.81 ± 0.71	0.89 (0.19, 1.58)	0.013	+0.45
Physical performance and clinical outcomes					
SPPB score	5.95 ± 2.39	6.09 ± 2.18	−0.15 (−1.17, 0.87)	0.775	−0.06
Five-times chair stand test (s)	17.54 ± 10.28	16.22 ± 9.59	1.32 (−3.10, 5.74)	0.555	+0.13
WOMAC total score (%)	63.01 ± 14.29	59.39 ± 16.78	3.62 (−3.09, 10.33)	0.286	+0.24
WOMAC pain score (%)	64.91 ± 15.30	60.78 ± 16.56	4.13 (−2.82, 11.08)	0.241	+0.26
Inflammatory and oxidative stress biomarkers					
CRP (mg/dL), median [IQR]	0.51 [0.19–0.93]	0.42 [0.16–0.67]	—	0.358 †	—
ln(CRP)	−0.60 ± 1.50	−0.83 ± 1.68	0.23 (−0.46, 0.93)	0.500	+0.15
NLR	2.92 ± 1.31	3.00 ± 2.35	−0.08 (−1.00, 0.85)	0.865	−0.04
Urinary F_2_-isoprostanes (ng/mg creatinine), median [IQR]	2.24 [1.68–2.83]	2.33 [1.62–3.05]	—	0.765 †	—
ln(Urinary F_2_-isoprostanes)	0.85 ± 0.59	0.79 ± 0.44	0.06 (−0.18, 0.30)	0.624	+0.11

Data are presented as mean ± SD unless otherwise indicated. CRP and urinary F_2_-isoprostanes are additionally presented in their original units as median [interquartile range (IQR)] to facilitate clinical interpretation. Natural logarithm-transformed values [ln(CRP) and ln(urinary F_2_-isoprostanes)] were used for parametric statistical analyses. † Between-group comparisons of the original, untransformed CRP and urinary F_2_-isoprostane values were performed using the Mann–Whitney U test. Mean differences and Hedges’ g are therefore not reported for these median [IQR] comparisons. OA: Osteoarthritis; SD: Standard Deviation; ASMM/H^2^: Appendicular Skeletal Muscle Mass adjusted for Height squared; SPPB, Short Physical Performance Battery; WOMAC, Western Ontario and McMaster Universities Osteoarthritis Index; CRP, C-reactive protein; NLR: Nneutrophil-to-Lymphocyte Ratio; and ln: natural logarithm.

**Table 2:** Distribution of EWGSOP2-defined sarcopenia phenotypes according to the affected joint.

EWGSOP2 Phenotype	Knee OA (*n* = 56)	Hip OA (*n* = 32)	Total (*n* = 88)	χ^2^	*p*-Value
Non-sarcopenic	39 (69.6%)	23 (71.9%)	62 (70.5%)	0.049	0.825
Probable/confirmed sarcopenia	17 (30.4%)	9 (28.1%)	26 (29.5%)		
Total	56 (100%)	32 (100%)	88 (100%)		

Data are presented as *n* (%). Differences between knee and hip OA groups were assessed using Pearson’s chi-square test. OA, osteoarthritis; EWGSOP2, European Working Group on Sarcopenia in Older People 2. The combined probable/confirmed sarcopenia group included 22 participants with probable sarcopenia and 4 participants with confirmed sarcopenia. All four participants with confirmed sarcopenia also fulfilled the criteria for severe sarcopenia.

**Table 3:** Comparison of body composition, muscle strength, physical performance, and inflammatory/oxidative stress biomarkers according to EWGSOP2 sarcopenia phenotype.

Variable	No Sarcopenia (*n* = 62) Mean ± SD	Probable/Confirmed Sarcopenia (*n* = 26) Mean ± SD	Mean Difference (95% CI)	*p*-Value	Hedges’ g
Body Composition ASMM/H^2^ (kg/m^2^)	9.32 ± 2.32	7.86 ± 1.98	1.46 (0.47, 2.45)	0.004	0.65
Body fat (%)	38.75 ± 10.76	45.06 ± 6.41	−6.31 (−10.37, −2.25)	0.001	0.67
Muscle strength and physical performance					
Handgrip strength (kg)	26.38 ± 11.93	11.36 ± 3.06	15.02 (10.45, 19.59)	<0.001	1.49
Gait speed (m/s)	0.87 ± 0.31	0.65 ± 0.26	0.22 (0.09, 0.35)	0.001	0.77
Inflammatory and oxidative stress biomarkers					
ln(CRP)	−0.74 ± 1.62	−0.53 ± 1.42	−0.21 (−0.91, 0.49)	0.550	0.13
NLR	2.99 ± 1.94	2.85 ± 1.18	0.14 (−0.56, 0.84)	0.683	0.08
ln(Urinary F_2_-isoprostanes)	0.73 ± 0.39	1.04 ± 0.75	−0.31 (−0.63, 0.004)	0.053	0.48

Data are presented as mean ± standard deviation (SD). Between-group comparisons were performed using independent-samples *t*-tests. Welch’s correction was applied when the assumption of homogeneity of variances was violated. Mean differences are presented as No sarcopenia − Probable/confirmed sarcopenia phenotype. Hedges’ g represents the standardized mean difference (bias-corrected effect size); values of approximately 0.2, 0.5, and ≥0.8 indicate small, moderate, and large effect sizes, respectively. Handgrip strength and ASMM/H^2^ are components of the EWGSOP2 classification algorithm; therefore, between-group differences in these variables should be interpreted as descriptive characteristics of the phenotype rather than independent associations with sarcopenia status. Abbreviations: EWGSOP2: European Working Group on Sarcopenia in Older People 2; ASMM/H^2^: appendicular skeletal muscle mass adjusted for height squared; CRP, C-reactive protein; NLR, neutrophil-to-lymphocyte ratio; Urinary F_2_-isoprostanes and CRP are presented as natural logarithm-transformed values [ln(urinary F_2_-isoprostanes); ln (CRP) respectively].

**Table 4:** Age-adjusted comparison of body composition, physical performance, and inflammatory/oxidative stress biomarkers according to EWGSOP2 sarcopenia phenotype.

Variable	No Sarcopenia Adjusted Mean (95% CI)	Probable/Confirmed Sarcopenia Adjusted Mean (95% CI)	Adjusted Mean Difference (95% CI)	F	*p*-Value	Partial η^2^
Body Composition						
Body fat (%)	39.61 (37.17, 42.05)	43.26 (39.41, 47.11)	−3.65 (−8.35, 1.05)	2.388	0.126	0.028
Physical Performance						
Gait speed (m/s)	0.83 (0.77, 0.90)	0.74 (0.63, 0.84)	0.10 (−0.03, 0.23)	2.223	0.140	0.026
Inflammatory and Oxidative Stress Biomarkers						
ln(CRP)	−0.81 (−1.22, −0.40)	−0.39 (−1.04, 0.26)	−0.42 (−1.21, 0.37)	1.113	0.295	0.013
NLR	3.02 (2.56, 3.47)	2.79 (2.06, 3.51)	0.23 (−0.65, 1.11)	0.267	0.606	0.003
ln(Urinary F_2_-isoprostanes)	0.77 (0.63, 0.90)	0.99 (0.78, 1.20)	−0.22 (−0.48, 0.04)	2.899	0.092	0.033

Abbreviations: EWGSOP2: European Working Group on Sarcopenia in Older People 2; CRP: C-reactive protein; NLR: neutrophil-to-lymphocyte ratio; Urinary F_2_-isoprostanes and CRP are presented as natural logarithm-transformed values [ln(urinary F_2_-isoprostanes); ln (CRP) respectively].

**Table 5:** Pearson correlation coefficients between muscle-related parameters, physical performance, inflammatory biomarkers, and oxidative stress biomarkers after BH-FDR correction.

Variable	ASMM/H^2^	ASMM/BMI	Handgrip	Chair Stand	Gait Speed	SPPB	Phase Angle	ln(CRP)	NLR	ln(Urinary F_2_-Isoprostanes)
ASMM/H^2^	—									
ASMM/BMI	0.738 *	—								
Handgrip strength	0.516 *	0.566 *	—							
Five-times chair stand test	−0.437 *	−0.462 *	−0.623 *	—						
Gait speed	0.419 *	0.475 *	0.635 *	−0.684 *	—					
SPPB score	0.397 *	0.446 *	0.539 *	−0.701 *	0.737 *	—				
Phase angle	0.448 *	0.473 *	0.491 *	−0.462 *	0.417 *	0.452 *	—			
ln(CRP)	−0.246 *	−0.181	−0.214	0.163	−0.223	−0.195	−0.187	—		
NLR	−0.232 *	−0.176	−0.138	0.119	−0.161	−0.144	−0.169	0.391 *	—	
ln(Urinary F_2_-isoprostanes)	−0.378 ***	−0.332 **	−0.391 *	0.324 **	−0.351 **	−0.298 **	−0.305 **	0.176	0.151	—

Data are Pearson correlation coefficients (*r*). Only the lower triangular correlation matrix is presented to improve readability. The Benjamini–Hochberg false discovery rate (BH-FDR) correction was applied across the 45 unique pairwise correlations to account for multiple testing. Correlation coefficients were interpreted as weak (*r* = 0.10–0.29), moderate (*r* = 0.30–0.49), and strong (*r* ≥ 0.50), as defined in the Statistical Analysis section. CRP and urinary F_2_-isoprostanes were natural logarithm-transformed before analysis. * BH-FDR-adjusted *p* < 0.05; ** BH-FDR-adjusted *p* < 0.01; *** BH-FDR-adjusted *p* < 0.001. Abbreviations: ASMM/H^2^: Appendicular Skeletal Muscle Mass adjusted for Height squared; ASMM/BMI: Appendicular Skeletal Muscle Mass adjusted for Body Mass Index; SPPB: Short Physical Performance Battery; CRP: C-Reactive Protein; NLR: Neutrophil-to-Lymphocyte Ratio; ln: natural logarithm.

**Table 6:** Hierarchical multiple linear regression analyses examining factors independently associated with Short Physical Performance Battery (SPPB) score.

Predictor	B (SE)	95% CI	β	*p*-Value
Model 1—Demographic variables				
Age	−0.070 (0.021)	(−0.112, −0.029)	−0.348	0.001
Gender	−0.040 (0.514)	(−1.062, 0.981)	−0.008	0.938
Model 1 statistics				
R^2^	0.129			
Adjusted R^2^	0.108			
Model F (*p*-value)	6.139 (0.003)			
df (Regression, Residual)	2, 83			
Model 2—Demographic and muscle-related variables				
Demographic variables				
Age	−0.047 (0.023)	(−0.090, −0.002)	−0.235	0.039
Sex	−0.593 (0.556)	(−1.700, 0.514)	−0.119	0.290
Muscle-related variables				
Handgrip strength	0.050 (0.024)	(0.003, 0.097)	0.264	0.039
ASMM/H^2^	0.074 (0.114)	(−0.153, 0.301)	0.074	0.520
Model 2 statistics				
R^2^	0.213			
Adjusted R^2^	0.174			
ΔR^2^	0.084			
Model F (*p*-value)	4.338 (0.002)			
df (Regression, Residual)	4, 81			

Data are presented as unstandardized regression coefficients (B) with standard errors (SE), standardized regression coefficients (β), 95% confidence intervals (CI), and *p* values. Model 1 included age and sex as demographic covariates. Model 2 additionally included handgrip strength and appendicular skeletal muscle mass adjusted for height squared (ASMM/H^2^). ΔR^2^ represents the increase in explained variance after the addition of the muscle-related variables in Model 2. Statistically significant associations (*p* < 0.05) are shown in bold. ASMM/H^2^, appendicular skeletal muscle mass adjusted for height squared; B, unstandardized regression coefficient; β, standardized regression coefficient; CI, confidence interval; SE, standard error; SPPB, Short Physical Performance Battery.

**Table 7:** Hierarchical multiple linear regression analyses examining factors independently associated with appendicular skeletal muscle mass adjusted for height squared (ASMM/H^2^).

Predictor	B (SE)	95% CI	β	*p*-Value
Model 1—Demographic variables				
Age	−0.016 (0.020)	(−0.055, 0.023)	−0.085	0.427
Gender	1.946 (0.503)	(0.946, 2.945)	0.391	<0.001
Model 1 statistics				
R^2^	0.172			
Adjusted R^2^	0.152			
Model F (*p*-value)	8.640 (<0.001)			
df (Regression, Residual)	2, 83			
Model 2—Demographic, inflammatory, and oxidative stress variables				
Demographic variables				
Age	−0.013 (0.019)	(−0.050, 0.024)	−0.070	0.490
Sex	1.922 (0.469)	(0.989, 2.856)	0.386	<0.001
Inflammatory and oxidative stress biomarkers				
ln(CRP)	−0.457 (0.214)	(−0.883, −0.031)	−0.230	0.037
ln(Urinary F_2_-isoprostanes)	−1.198 (0.409)	(−2.011, −0.385)	−0.282	0.005
Model 2 statistics				
R^2^	0.295			
Adjusted R^2^	0.261			
ΔR^2^	0.123			
Model F (*p*-value)	8.671 (<0.001)			
df (Regression, Residual)	4, 81			
Model 3—Additional adjustment for NLR				
Demographic variables				
Age	−0.013 (0.019)	(−0.050, 0.025)	−0.069	0.496
Sex	1.914 (0.472)	(0.975, 2.853)	0.385	<0.001
Inflammatory and oxidative stress biomarkers				
ln(CRP)	−0.358 (0.243)	(−0.841, 0.125)	−0.180	0.144
ln(Urinary F_2_-isoprostanes)	−1.147 (0.425)	(−1.991, −0.303)	−0.265	0.008
Additional inflammatory biomarker				
NLR	−0.071 (0.127)	(−0.324, 0.181)	−0.060	0.576
Model 3 statistics				
R^2^	0.298			
Adjusted R^2^	0.255			
ΔR^2^	0.003			
Model F (*p*-value)	6.906 (<0.001)			
df (Regression, Residual)	5, 80			

Data are presented as unstandardized regression coefficients (B) with standard errors (SE), standardized regression coefficients (β), 95% confidence intervals (CI), and *p* values. Model 1 included age and sex as demographic covariates. Model 2 additionally included ln-transformed (CRP) and ln-transformed urinary F_2_-isoprostanes. Model 3 included the neutrophil-to-lymphocyte ratio (NLR). ΔR^2^ represents the increase in explained variance relative to the preceding hierarchical model. Statistically significant associations (*p* < 0.05) are shown in bold. Abbreviations: ASMM/H^2^: Appendicular Skeletal Muscle Mass adjusted for Height squared; B: unstandardized regression coefficient; β: standardized regression coefficient; CI: confidence interval; CRP: C-reactive protein; NLR: neutrophil-to-lymphocyte ratio; and SE: Standard Error.

**Table 8:** Hierarchical multiple linear regression analyses examining factors independently associated with appendicular skeletal muscle mass adjusted for body mass index (ASMM/BMI).

Predictor	B (SE)	95% CI	β	*p*-Value
Model 1—Demographic variables				
Age	−0.008 (0.002)	(−0.012, −0.005)	−0.402	<0.001
Sex	0.211 (0.049)	(0.113, 0.309)	0.338	<0.001
Model 1 statistics				
R^2^	0.381			
Adjusted R^2^	0.366			
Model F (*p*-value)	26.159 (<0.001)			
df (Regression, Residual)	2, 85			
Model 2—Demographic variables and oxidative stress				
Demographic variables				
Age	−0.008 (0.002)	(−0.012, −0.005)	−0.397	<0.001
Sex	0.205 (0.048)	(0.110, 0.300)	0.329	<0.001
Oxidative stress biomarker				
ln(Urinary F_2_-isoprostanes)	−0.082 (0.038)	(−0.158, −0.007)	−0.188	0.034
Model 2 statistics				
R^2^	0.414			
Adjusted R^2^	0.393			
ΔR^2^	0.033			
Model F (*p*-value)	19.782 (<0.001)			
df (Regression, Residual)	3, 84			
Model 3—Additional adjustment for NLR				
Demographic variables				
Age	−0.008 (0.002)	(−0.012, −0.004)	−0.392	<0.001
Sex	0.203 (0.050)	(0.104, 0.302)	0.324	<0.001
Oxidative stress biomarker				
ln(Urinary F_2_-isoprostanes)	−0.076 (0.040)	(−0.155, 0.004)	−0.174	0.060
Inflammatory biomarker				
NLR	−0.027 (0.018)	(−0.063, 0.009)	−0.131	0.136
Model 3 statistics				
R^2^	0.430			
Adjusted R^2^	0.402			
ΔR^2^	0.016			
Model F (*p*-value)	15.654 (<0.001)			
df (Regression, Residual)	4, 83			

Data are presented as unstandardized regression coefficients (B) with standard errors (SE), standardized regression coefficients (β), 95% confidence intervals (CI), and *p* values. Model 1 included age and sex as demographic covariates. Model 2 additionally included ln-transformed urinary F_2_-isoprostanes. Model 3 additionally included the neutrophil-to-lymphocyte ratio (NLR). ΔR^2^ represents the increase in explained variance relative to the preceding hierarchical model. Statistically significant associations (*p* < 0.05) are shown in bold. Abbreviations: ASMM/BMI, appendicular skeletal muscle mass adjusted for body mass index; B, unstandardized regression coefficient; β, standardized regression coefficient; CI, confidence interval; NLR, neutrophil-to-lymphocyte ratio; and SE, standard error.

**Table 9:** Medication intake according to Dyslipidaemia Status.

Medication Use	No Dyslipidaemia *(n* = 56)	Dyslipidaemia (*n* = 32)	Total (N = 88)	*p*-Value
Antidiabetic medication	8 (14.3%)	2 (6.3%)	10 (11.4%)	0.316
Statins / lipid-lowering medication	0 (0.0%)	32 (100.0%)	32 (36.4%)	<0.001
Antihypertensive medication	30 (53.6%)	15 (46.9%)	45 (51.1%)	0.658
NSAID use for pain relief	42 (75.0%)	27 (84.4%)	69 (78.4%)	0.421
Corticosteroid medication	1 (1.8%)	0 (0.0%)	1 (1.1%)	1.000

Data are presented as *n* (%), with percentages calculated within each dyslipidaemia group. NSAID use refers to documented use of NSAIDs for pain relief on an as-needed basis and does not indicate regular or continuous treatment. Dyslipidaemia was coded as 0 = no and 1 = yes. Comparisons between groups were conducted using Fisher’s exact test.

**Table 10:** Exploratory comparison of body composition, muscle-related parameters, inflammatory biomarkers, and oxidative stress biomarkers according to dyslipidemia status.

Variable	No Dyslipidemia (*n* = 56) Mean ± SD	Dyslipidemia (*n* = 32) Mean ± SD	Mean Difference (95% CI)	Hedges’ g	*p*-Value
Body composition					
Body fat (%)	38.62 ± 11.11	44.15 ± 6.88	−5.53 (−9.83, −1.23)	0.55	0.014
ASMM/H^2^ (kg/m^2^)	9.04 ± 2.33	8.53 ± 2.12	0.51 (−0.47, 1.49)	0.23	0.305
ASMM/BMI	0.76 ± 0.25	0.66 ± 0.18	0.10 (0.003, 0.189)	0.43	0.044
Appendicular skeletal muscle mass (ASMM, kg)	24.00 ± 7.71	21.90 ± 5.71	2.10 (−1.06, 5.26)	0.30	0.190
Lean mass (kg)	50.52 ± 14.65	47.06 ± 12.16	3.46 (−2.74, 9.66)	0.25	0.268
Phase angle (°)	5.60 ± 2.35	5.01 ± 1.02	0.59 (−0.31, 1.49)	0.30	0.198
Inflammatory and oxidative stress biomarkers					
ln(CRP)	−0.59 ± 1.63	−0.98 ± 1.25	0.39 (−0.29, 1.07)	0.26	0.260
NLR	2.76 ± 1.29	2.92 ± 1.19	−0.16 (−0.73, 0.41)	0.13	0.585
ln(Urinary F_2_-isoprostanes)	0.90 ± 0.53	0.67 ± 0.53	0.23 (0.001, 0.459)	0.43	0.049
Physical performance and clinical outcomes					
SPPB score	6.21 ± 2.40	5.65 ± 2.15	0.56 (−0.45, 1.57)	0.24	0.275
WOMAC total score (%)	59.13 ± 14.93	65.70 ± 14.95	−6.57 (−13.25, 0.11)	0.44	0.053

Data are presented as mean ± standard deviation (SD). Between-group comparisons were performed using independent-samples *t*-tests. Mean differences are presented as No dyslipidemia − Dyslipidemia. Hedges’ g represents the bias-corrected standardized mean difference; values of approximately 0.2, 0.5, and ≥0.8 indicate small, moderate, and large effect sizes, respectively. Statistically significant differences (*p* < 0.05) are shown in bold. Abbreviations: ASMM, appendicular skeletal muscle mass; ASMM/H^2^, appendicular skeletal muscle mass adjusted for height squared; ASMM/BMI, appendicular skeletal muscle mass adjusted for body mass index; CRP, C-reactive protein; NLR, neutrophil-to-lymphocyte ratio; SPPB, Short Physical Performance Battery; WOMAC, Western Ontario and McMaster Universities Osteoarthritis Index.

## Data Availability

The data presented in this study are not publicly available due to the sensitive nature of the participants’ clinical information and because of the strict ethical restrictions regarding privacy of patients. Furthermore, the data is part of an ongoing research project with additional planned analysis. Anonymized data can be requested from the corresponding authors upon reasonable request.
